# Autophagy Maintains the Function of Bone Marrow Mesenchymal Stem Cells to Prevent Estrogen Deficiency-Induced Osteoporosis: Erratum

**DOI:** 10.7150/thno.81713

**Published:** 2023-07-26

**Authors:** Meng Qi, Liqiang Zhang, Yang Ma, Yi Shuai, Liya Li, Kefu Luo, Wenjia Liu, Yan Jin

**Affiliations:** 1 State Key Laboratory of Military Stomatology & National Clinical Research Center for Oral Diseases & Shaanxi International Joint Research Center for Oral Diseases, Center for Tissue Engineering, School of Stomatology, Fourth Military Medical University, Xi'an, China;; 2 Xi'an Institute of Tissue Engineering and Regenerative Medicine, Xi'an, China;; 3 State Key Laboratory of Military Stomatology & National Clinical Research Center for Oral Diseases & Shaanxi Clinical Research Center for Oral Diseases, Department of Prosthodontics, School of Stomatology, The Fourth Military Medical University, Xi'an, China.

Oil Red O staining images of bone marrow cavities in Figure 6D may have been chosen incorrectly due to incorrect data naming. We have repeated the experiment in order to ensure that this work is scientifically rigorous. Following is a revised arrangement of Figure 6D. It is noteworthy that the correction has no effect on our conclusion. Please accept our sincere apologies to the Editor and the readership of the journal for any inconvenience caused.

## Figures and Tables

**Figure 6:**
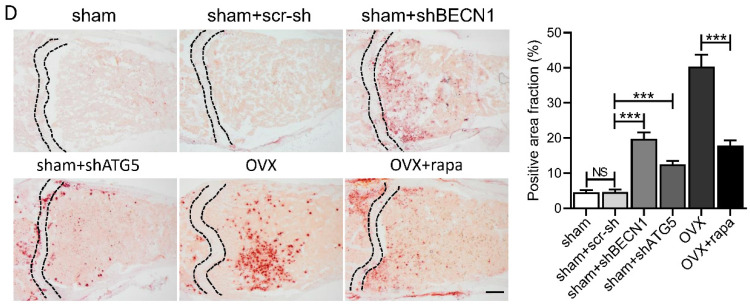
** Autophagy level controls the skeletal phenotype in mice.** (D) Bone marrow cavity were stained with Oil Red O to calculate the percentage of adipocytes in areas under growth plates of distal femur (n=5). Scale bar, 200 μm. Data are shown as mean ± SD, *P < 0.05, **P < 0.01, ***P < 0.001.

